# Gastric cancer clinical characteristics and their altered trends in South China: An epidemiological study with 2,800 cases spanning 26 years

**DOI:** 10.3389/fonc.2023.976854

**Published:** 2023-02-07

**Authors:** Hongfa Wei, Xiao-Yong Zhan, Xianying Liao, Wenchao Li, Hui Chen, Cuncan Deng, Xinghan Jin, Zhangsen Huang, Mo Yang, Changhua Zhang, Yulong He

**Affiliations:** ^1^ Digestive Diseases Center, The Seventh Affiliated Hospital of Sun Yat-sen University, Shenzhen, China; ^2^ Scientific Research Center, The Seventh Affiliated Hospital, Sun Yat-sen University, Shenzhen, China; ^3^ Invasive Technology Department of the Cancer Hospital of Shantou University Medical College, Shantou, China

**Keywords:** gastric cancer, epidemiology, South China, trend, clinical characteristics

## Abstract

**Background:**

Gastric cancer (GC) is a serious threat to human health. The clinical GC characteristics in China may be impacted by changes in people’s lifestyles and the promotion of early GC (EGC) screening. The present study aims to evaluate the recent trends of GC characteristics in South China and search for hazardous factors limiting the survival time of GC patients.

**Methods:**

Data on GC patients that were hospitalized in the Department of Digestive Center, the First Affiliated Hospital, Sun Yat-sen University, from 1994 to 2019 were collected and divided into two categories according to the time when the EGC screening began in China: the PRE group (previous 13 years, 1994–2006) and the PAS group (past 13 years, 2007–2019).

**Results:**

We found that, although the 5-year survival rate increased in the PAS group compared with the PRE group (*P* < 0.0001), patients with age ≥60 years or Borrmann type IV still had a worse prognosis. In the PAS group, the larger percentages of elderly patients and patients with Borrmann type IV in the lymphatic metastases (N1) group (41.0% *vs*. 51.1%, *P* = 0.0014) and stage IV subgroup (20.7% *vs*. 32.2%, *P* = 0.016), respectively, when compared with the PRE group, may have contributed to the poor outcome of GC. By comparing the odds ratio (OR) of 5-year overall survival (OS) in the two 13-year periods, female sex and T2 turned into risk factors because of a greater proportion of Borrmann type IV or elderly patients in the PAS group (OR = 0.983, 95% CI = 0.723–1.336 *vs*. OR = 1.277, 95% CI = 1.028–1.586 and OR = 1.545, 95% CI = 0.499–4.775 *vs*. OR = 2.227, 95% CI = 1.124–4.271, respectively).

**Conclusions:**

Despite the GC epidemiology changes, the overall prognosis of GC patients has improved in South China. However, old age and Borrmann type IV are still the major restrictions affecting the survival of GC patients, a situation which calls for additional attention.

## 1 Introduction

Gastric cancer (GC) is the sixth most common cancer worldwide, with over 1 million estimated new cases annually. In 2020, more than 0.7 million people died from GC in the whole world, making it the fourth most common cause of cancer-related death (1). According to the Global Burden of Disease Study (GBD) 2017 General Commentary, the age-standardized incidence of mortality is on the decline globally, with 28% in 2017 and 48.7% in 1990, particularly in industrialized Asian nations like Japan and South Korea (2). However, the number of instances of GC is still increasing (2). The common symptoms of GC are dyspepsia, anorexia, or early satiety, weight loss, and abdominal pain, which make it difficult to diagnose. The 5-year overall survival (OS) rate for advanced GC was formerly believed to be about 30%, but 90% for early gastric cancer (EGC) (3, 4). Therefore, EGC screening is essential for enhancing the GC patients’ prognoses. In East Asia, the high-incidence area of GC, particularly China, the measures of EGC screening, such as routine gastroscopy and upper gastrointestinal series, significantly increase the survival of GC patients (5–7). Numerous studies have noted that GC patients typically have a narrow age range, and if diagnosed at an earlier clinical stage, they will have a better prognosis (5, 7, 8). Recently, clinicians have paid close attention to the trends in GC clinical parameters, including demographics, pathological kinds, and stages, particularly in terms of risk factors (9, 10). They tried to determine how demographic, clinical, and histological factors affect GC survival and concentrate on those risk factors to treat GC more effectively. However, the corresponding alterations stratified by certain clinicopathological parameters, such as hospitalization, Borrmann type, tumor site, pTNM, histological type, and their relationships, were not fully comparable in those investigations. Therefore, a detailed and large-scale analysis of the clinical features and the altering trends of GC in China is required to guide future GC control strategies (11). Our goal was to evaluate the recent trends of GC characteristics in South China according to the clinicopathological factors of patients in 1994–2006 and 2007–2019.

## 2 Materials and methods

### 2.1 Ethics statement

The study was approved by the Ethics Committee of the Seventh Affiliated Hospital, Sun Yat-sen University. No informed consent from patients was required. No personal identification data or potentially identifiable images were included in the study.

### 2.2 Data sources

A total of 2,804 cases with preoperative GC diagnosed at Sun Yat-sen University’s First Affiliated Hospital between 1994 and 2019 were enrolled to characterize the distinct clinicopathological characteristics and prognosis of GC patients in South China. These cases were split into two groups based on the First Affiliated Hospital’s introduction of EGC screening in 2007: the previous 13-year group (the PRE group, diagnosed between January 1994 and December 2006, *n* = 766) and the past 13-year group (the PAS group, diagnosed between January 2007 and December 2019, *n* = 2038). The inclusion criteria of the GC cases were as follows (1): primary GC that had surgery, (2) detailed pathological report was available, and (3) no chronic and severe diseases in the major organs (kidney, liver, heart, *etc.*). The exclusion criteria were as follows: (1) patients with gastroesophageal junction tumors, gastric carcinoid, gastric lymphoma, and gastrointestinal stromal tumors, (2) incomplete clinical and pathological information and missing survival data, (3) history of other malignant tumors, and (4) stage 0 GC was confirmed pathologically based on the American Joint Committee on Cancer (AJCC) staging system.

### 2.3 Diagnostic criteria

Anatomically, the stomach is split into three parts: gastric cardia, corpus, and antrum. The term “the whole gastric cancer” was used if the tumor has invaded every gastric area. All patients were subjected to thorax and abdomen CT scan with oral and IV contrast and barium-meal joint with a gastroscope in the upper gastrointestinal tract to locate the tumor size. The endoscopic optical biopsy was used for GC diagnosis *in vivo*. The macroscopic classification of GC was classified according to Borrmann as type I (polypoid without ulceration and broad base), type II (ulcerated with elevated borders and sharp margins), type III (ulcerated with diffuse infiltration at the base), and type IV (diffusely infiltrative thickening of the wall) (12). The classification of histological type was according to the criteria of the World Health Organization (13). The tumor differentiation is classified as well-differentiated type, moderately differentiated type, and poorly differentiated type. The stage is classified according to the seventh edition of the AJCC staging system.

### 2.4 Statistical analysis

Continuous variables were presented as medians with interquartile ranges. Categorical variables were calculated for the frequency in each category. The survival rates were estimated by the Kaplan–Meier method, and log-rank test was used to identify differences between the survival curves. Chi-square tests and nonparametric Mann–Whitney *U*-tests were used to assess categorical covariates and continuous variables, respectively. Chi-square tests were also utilized to analyze the clinicopathological information in univariate analysis, while Cox’s proportional hazard model for risk factors with prognosis was used in the multivariate analysis. Pearson correlation coefficient was used to display the relationship between patients’ population of all diseases from Guangzhou and the GC patients from our study. Kendall’s Tau-b correlation analysis was used to analyze the relationship among differently ranked indices. The relative risk (RR) with a corresponding 95% confidence interval (CI) was calculated using a random-effects model. *P <*0.05 was set to be statistically significant. Statistical analyses were performed by using SPSS 22.0 (SPSS Inc., Chicago, IL, USA). Graphing was performed with GraphPad Prism 8.0.2 (GraphPad Software).

## 3 Results

### 3.1 Changes in the clinical and pathologic features of GC patients in South China

**Figure 1:**
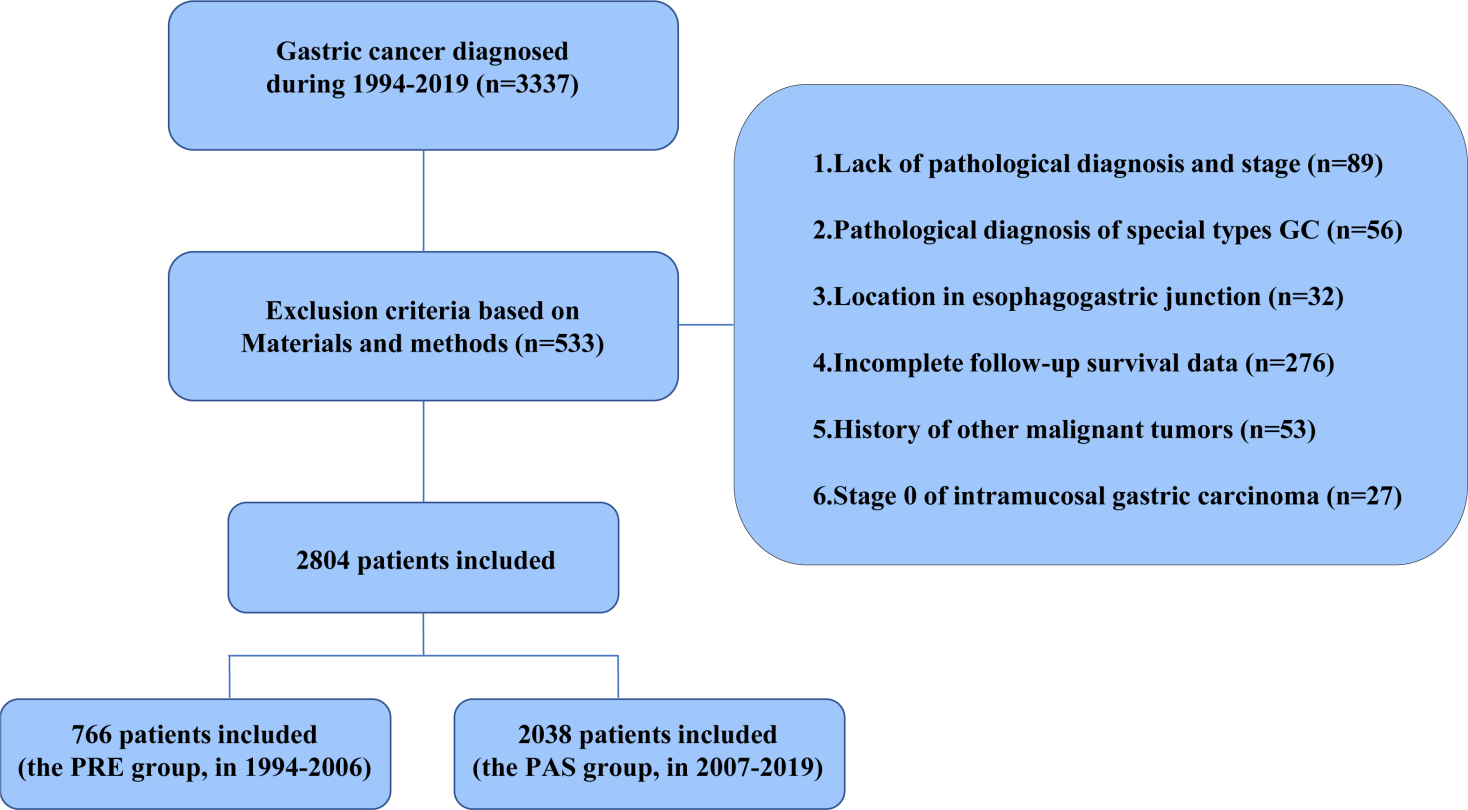
Flow chart of the selection of gastric cancer patients in the database.

The flow chart in **Figure 1** shows that 2,804 patients with GC were enrolled based on the exclusion criteria. Guangzhou is one of the most emblematic cities in South China, where the First Affiliated Hospital, Sun Yat-sen University, is a top-tier, contemporary, Grade A general hospital in China, ranked sixth in China and first in South China. Upon consultation, the Guangzhou Statistical yearbook (1994–2019) shows that there is no significant difference between the proportion of GC patients grouped in our study and the proportion of patients in Guangzhou (**Supplementary Figure S1A**). In comparison with the PRE group, the patient population size of the PAS group expanded from 2007 to 2019 and was correlated with the number of patients with all diseases in Guangzhou (**Supplementary Figure S1B**). The number of patients in our study and the number of patients in Guangzhou are strongly positively correlated (*R* = 0.8946, *P* < 0.0001, **Supplementary Figure S1C**). Consequently, the GC patients enrolled in our study could represent those in Southern China.

The demography of patient characteristics is shown in **Table 1**. The proportion of young patients (<40 years old) was lower in the PAS group, while the proportion of elderly ones (≥60 years old) had no change (11.6% *vs*. 8.5%, *P* = 0.0112; 45.4% *vs*. 49.0%, *P* = 0.0902, **Table 1**). In the PAS group, the post-operative patients had a shorter hospital stay (13 *vs*. 10 days, *P* < 0.0001, **Table 1**). More cases stayed in the hospital for less than 8 days after surgery (3.7% *vs*. 33.1%, *P* < 0.0001, **Table 1**). The tumors in the patients of the PAS group were smaller than those in the PRE group (3.5 *vs*. 5.0 cm, *P* < 0.0001, **Table 1**). The proportion of peritoneal and liver metastasis was becoming comparably lower (14.8% *vs*. 12.3% and 5.5% *vs*. 2.1%, *P* = 0.0133 and *P* < 0.0001, respectively; **Table 1**). The tumor was found to become more frequently located in just only a single part of the stomach (70.8% *vs*. 75.9%, *P* = 0.0074, **Table 1**). T4, which means the tumor invades the serosa (visceral peritoneum) or adjacent tissues, was classified much less frequently than the other T stages (55% *vs*. 18.3%, *P* < 0.0001, **Table 1**), and the proportion of GC patients with pathologic stage (pTNM) IV became lower compared with the patients in the PRE group (27.8% *vs*. 19.5%, *P* < 0.0001, **Table 1**). Some results were beyond our expectations. Firstly, Borrmann type I became less diagnosed (6.2% *vs*. 3.3%, *P* = 0.0011, **Table 1**), while the proportion of Borrmann type IV was unchanged (12.0% *vs*. 12.8%, *P* = 0.2190, **Table 1**). Secondly, well-differentiated GC came to have lesser incidence than the other two histological types (5.2% vs.1.9%, *P* < 0.0001, **Table 1**). Finally, there was no significant difference in the proportions of lymphatic metastasis (N stage) between the patients of the two groups (65.4% *vs*. 62.4%, *P* = 0.2190, **Table 1**).

**Table 1:** Demographics and characteristics of the gastric cancer patients in the PRE group and the PAS group.

Characteristics	Total	1994–2006 (PRE group)	2007–2019 (PAS group)	*P*-value
Proportion of patients	2,804	766 (27.3%)	2,038 (72.7%)	
Demographic				
Survival (months)	24.9 (10.6–59.0)	31.5 (11.7–135.4)	22.6 (10.2–50.8)	**<0.0001** ^a^
Survival or death rate (*n*)				
Alive	1,423	188 (24.5%)	1,235 (60.6%)	
Dead	1,381	578 (75.5%)	803 (39.4%)	
Age, years	59.0(50.0–66.0)	58.0(48.0–66.0)	59.0(51.0–66.0)	**0.0055** ^a^
<40	262	89 (11.6%)	173 (8.5%)	**0.0112** ^b^
40–49	420	131 (17.1%)	289 (14.2%)	
50–59	775	198 (25.8%)	577 (28.3%)	
≥60	1,347	348 (45.4%)	999 (49.0%)	0.0902^c^
Gender				
Male, *n* (%)	1,835	502 (65.5%)	1,333 (65.4%)	0.9496^d^
Female, *n* (%)	969	264 (34.5%)	705 (34.6%)	
Hospitalization, days	11.0 (8.0–14.0)	13.0(11.0–18.0)	10.0(8.0–13.0)	**<0.0001** ^a^
≤8, *n* (%)	703	28 (3.7%)	675 (33.1%)	**<0.0001** ^e^
9–15, *n* (%)	1,514	479 (62.5%)	675 (50.8%)	
>15, *n* (%)	587	259 (33.8%)	328 (16.1%)	
Peritoneal metastasis, *n* (%)				
P0	2,453	652 (85.2%)	1,801 (87.7%)	**0.0133** ^d^
P1	343	113 (14.8%)	230 (12.3%)	
Hepatic metastasis, *n* (%)				
H0	2,713	723 (94.5%)	1,990 (97.9%)	**<0.0001** ^d^
H1	84	42 (5.5%)	42 (2.1%)	
Tumor location, *n* (%)				
A single part of the stomach	2,014	517 (70.8%)	1,497 (75.9%)	**0.0074** ^f^
Two parts of the stomach	548	166 (22.7%)	382 (19.4%)	
The whole stomach	141	47 (6.4%)	94 (4.8%)	
Tumor size (cm)	4 (3.0–6.0)	5.0 (3.5–7.0)	3.5 (2.0–5.0)	**<0.0001** ^a^
Borrmann type				
Type I	107	46 (6.2%)	61 (3.3%)	**0.0011** ^g^
Type II	615	175 (23.5%)	440 (24.1%)	
Type III	1,524	434 (58.3%)	1,090 (59.8%)	
Type IV	322	89 (12.0%)	233 (12.8%)	0.5731^h^
Histological type				
Well differentiated	68	38 (5.2%)	30 (1.9%)	**<0.0001** ^i^
Moderately differentiated	540	162 (22.2%)	378 (23.4%)	
Poorly differentiated	1,736	530 (72.6%)	1,206 (74.7%)	
Pathological type				
Adenocarcinoma	1,882	631 (91.3%)	1,251 (92.6%)	0.308
Others	160	60 (8.7%)	100 (7.4%)	
T stage				
T1 + T2 + T3	1,868	327 (45.0%)	1,541 (81.7%)	**<0.0001** ^d^
T4	745	400 (55.0%)	345 (18.3%)	
N stage	2.0 (0–8.0)	2.0 (0–8.0)	2.0 (0–8.0)	0.7856
N0	930	165 (34.6%)	765 (37.6%)	0.2190^d^
N1	1,581	312 (65.4%)	1,269 (62.4%)	
pTNM				
Stage: I + II + III	1,938	332 (72.2%)	1,606 (80.5%)	**<0.0001** ^d^
Stage: IV	517	128 (27.8%)	389 (19.5%)	

a Continuous variables are presented as medians with interquartile ranges, and nonparametric Mann–Whitney U-tests were used in the calculation. Other variables are categorical variables, and chi-square tests were used in the calculation.

b Patients younger than 40 years or older than 40 years were compared.

c Patients older than 60 years or younger than 60 years were compared.

d Chi-square tests were used to compare two 13-year-period patients with different indices.

e The time of hospitalization which was less than 8 days was compared with the hospital time which was more than 8 days.

f The tumor located in one part was compared with the tumor that invaded more than one part.

g Borrmann type I of gastric cancer was compared with the other Borrmann types.

h Borrmann type IV of gastric cancer was compared with the other Borrmann types.

i The tumor which was classified as well differentiated was used to compare with the other groups.

The bold values means significant meanings.

### 3.2 Survival outcomes have been significantly improved in GC patients

**Figure 2:**
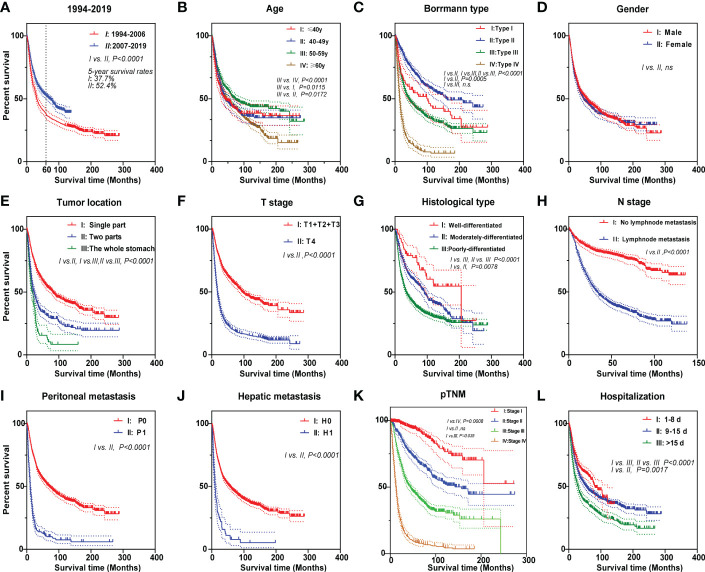
Kaplan–Meier survival curves of gastric cancer patients for different prognostic factors. The curves according to **(A)** two 13-year periods, **(B)** age, **(C)** Borrmann type, **(D)** gender, **(E)** tumor location, **(F)** T stage, **(G)** histological type, **(H)** N stage, **(I)** peritoneal metastasis, **(J)** hepatic metastasis, **(K)** pTNM, and **(L)** hospitalization. Log-rank was used to compare the curves.

The median follow-up period was 24.9 months in the 26 years (**Table 1**). We are concerned that patients in the PAS group experienced greater overall patient survival rates (*P* < 0.0001, **Figure 2A**). The overall 5-year survival rate in the PAS group was higher than in the PRE group (52.4% *vs*. 37.7%, **Figure 2A**). Patients diagnosed between 2010 to 2014 had an overall 5-year survival rate of 52.0% (**Supplementary Figure S1D**), which was lower than that of Japan and South Korea over the same period (14). Patients with ages more than 60 years showed a worse prognosis (**Figure 2B**; **Supplementary Figure S1E**). Borrmann type II had the best survival outcome (**Figure 2C**). Male and female patients had similar prognoses (**Figure 2D**). As expected, patients with gastric tumors limited to a single location, tumor invasion ≤T3, well differentiation, tumor without lymphatic metastasis, peritoneal metastasis, hepatic metastasis, and stage I all had better prognosis (**Figures 2E–K**). Some results were attracting our attention. Firstly, the prognosis was best in the patients aged 50–59 years (**Figure 2B**). Further analysis indicated that fewer Borrmann type IV patients were occupied in the GC patients aged 50–59 years (**Supplementary Table S1**). Secondly, the best prognosis for post-operative patients had a stay of 1 and 8 days in the hospital, while the poorest prognosis had a stay of more than 15 days (**Figure 2L**). Finally, the survival rate of patients with hepatic metastasis, peritoneal metastasis, stage IV, and lymphatic metastasis (N1) had no significant difference in the two groups (**Supplementary Figures S1F–I**). This might be related to the higher proportions of old patients (≥60 years) in the N1 subgroup (41% *vs*. 51.1%, *P* = 0.0014, **Table 2**) and Borrmann type IV in the stage IV subgroup (20.7% *vs*. 32.2%, *P* = 0.016, **Table 2**) of the PAS group. The **Supplementary Figure S2** and **Supplementary Figure S3** images illustrate how the prognosis of subgroups in the PAS group significantly improved when compared with those in the PRE group.

**Table 2:** Demographics and characteristics of patients in the N1 group and the stage IV group.

Characteristics	Lymphatic metastasis (N1)						Stage IV					
	Total	1994–2006		2007–2019		*P*-value	Total	1994–2006		2007–2019		*P*-value
Proportion of patients	1,581	312	(19.7%)	1,269	(80.3%)		517	128	(24.8%)	389	(75.2%)	
Demographic												
Age, years												
<40	149	42	(13.5%)	107	(8.4%)		51	16	(12.5%)	35	(9.0%)	
40–49	237	59	(18.9%)	178	(14.0%)		102	30	(23.4%)	72	(18.5%)	
50–59	418	83	(26.6%)	335	(26.4%)		137	32	(25.0%)	105	(27.0%)	
≥60	777	128	(41.0%)	649	(51.1%)	**0.0014**	227	50	(39.1%)	177	(45.5%)	0.2029
Gender												
Male, n (%)	1,022	201	(64.4%)	821	(64.7%)	0.947	331	76	(59.4%)	255	(65.6%)	0.243
Female, n (%)	559	111	(35.6%)	448	(35.3%)		186	52	(40.6%)	134	(34.4%)	
Hospitalization, days												
≤8, *n* (%)	407	16	(5.1%)	391	(30.8%)		99	8	(6.2%)	91	(23.4%)	
9–15, *n* (%)	865	202	(64.7%)	663	(52.2%)		274	73	(57.0%)	201	(51.7%)	
>15, *n* (%)	309	94	(30.1%)	215	(16.9%)	**<0.0001**	144	47	(36.7%)	97	(24.9%)	**0.0099**
Peritoneal metastasis, *n* (%)												
P0	1,337	245	(78.8%)	1,092	(86.1%)	**0.002**	287	74	(57.8%)	213	(55.2%)	0.61
P1	242	66	(21.2%)	176	(13.9%)		227	54	(42.2%)	173	(44.8%)	
Hepatic metastasis, *n* (%)												
H0	1,537	294	(94.5%)	1,243	(98.0%)	**0.002**	466	112	(87.5%)	354	(91.5%)	
H1	42	17	(5.5%)	25	(2.0%)		49	16	(12.5%)	33	(8.5%)	0.223
Tumor location, *n* (%)												
A single part of the stomach	1,106	213	(71.7%)	893	(72.2%)		272	77	(63.1%)	195	(53.6%)	
Two parts of the stomach	331	60	(20.2%)	271	(21.9%)		158	32	(26.2%)	126	(34.6%)	
The whole stomach	97	24	(8.1%)	73	(5.9%)	0.166	56	13	(10.7%)	43	(11.8%)	0.729
Borrmann type												
Type I	40	7	(2.3%)	33	(2.8%)		18	8	(6.7%)	10	(3.0%)	
Type II	220	39	(13.1%)	181	(15.2%)		25	12	(9.9%)	13	(3.8%)	
Type III	1,015	218	(73.2%)	797	(67.1%)		282	76	(62.8%)	206	(60.9%)	
Type IV	210	34	(11.4%)	176	(14.8%)	0.131	134	25	(20.7%)	109	(32.2%)	**0.016**
Histological type												
Well differentiated	11	8	(2.6%)	3	(0.3%)		2	2	(1.6%)	0	(0.0%)	
Moderately differentiated	252	60	(19.4%)	192	(18.2%)		68	18	(14.6%)	50	(15.7%)	
Poorly differentiated	1,099	241	(78.0%)	858	(81.5%)	0.172	372	103	(83.7%)	269	(84.3%)	0.879
Pathological type												
Adenocarcinoma	1,049	256	(92.8%)	793	(91.9%)	0.6429	324	109	(92.4%)	215	(90.3%)	0.5271
Others	90	20	(7.2%)	70	(8.1%)		32	9	(7.6%)	23	(9.7%)	
T stage												
T1 + T2 + T3	1,094	147	(47.3%)	947	(77.8%)		184	39	(33.1%)	145	(41.0%)	
T4	435	164	(52.7%)	271	(22.2%)	**<0.0001**	288	79	(66.9%)	209	(59.0%)	0.127
N stage												
N0							88	16	(16.2%)	72	(18.7%)	
N1							397	83	(83.8%)	314	(81.3%)	0.567
Stage												
Stage: I + II + III	1,133	180	(68.4%)	953	(75.2%)							
Stage: IV	397	83	(31.6%)	314	(24.8%)	**0.023**						

Categorical variables are calculated for the frequency in each category, and chi-square tests were used in the calculation.

The bold values means significant meanings.

### 3.3 Risk factors associated with the fatal outcome of GC

Risk factors associated with GC were evaluated through univariable- and multivariable-adjusted Cox proportional hazard model analysis. The factors, including peritoneal metastasis, hepatic metastasis, tumor invading the whole stomach, Borrmann type IV, deeper tumor invading, poor pathological stage, and lymphatic metastasis, were all identified as independent predictors of poor overall survival (**Table 3**). The period (the PAS group) was a protective factor in the multivariable analysis (hazard ratio, HR: 0.794, 95% CI = 0.666–0.946, *P* = 0.010, **Table 3**). Similarly, the patients’ age range of 50–59 was an independent protective factor for a better outcome (HR: 0.772, 95% CI = 0.601-0.992, *P* = 0.043, **Table 3**).

**Table 3:** Univariable and multivariable COX analyses of gastric cancer characteristics.

1994–2019	Univariate analysis			Multivariate analysis		
Characteristics	Hazard ratio	95% confidence interval	*P*-value	Hazard ratio	95% confidence interval	*P*-value
Years						
1994–2006	Reference			Reference		
2007–2019	0.702	(0.628–0.784)	**<0.0001**	0.794	(0.666–0.946)	**0.010**
Age						
<40	Reference			Reference		
40–49	0.955	(0.770–1.183)	0.671	0.845	(0.649–1.100)	0.212
50–59	0.78	(0.639–0.952)	**0.015**	0.772	(0.601–0.992)	**0.043**
≥60	1.048	(0.872–1.259)	0.618	1.044	(0.826–1.320)	0.716
Gender						
Male, *n* (%)	Reference			Reference		
Female, *n* (%)	1.047	(0.938–1.170)	0.413	1.051	(0.911–1.213)	0.497
Hospitalization						
≤8, *n* (%)	Reference			Reference		
9–15, *n* (%)	1.262	(1.086–1.468)	**0.002**	0.901	(0.750–1.082)	0.265
>15, *n* (%)	1.859	(1.575–2.196)	**<0.0001**	1.136	(0.915–1.412)	0.248
Peritoneal metastasis						
P0	Reference			Reference		
P1	3.999	(3.504–4.564)	**<0.0001**	1.425	(1.173–1.731)	**<0.0001**
Hepatic metastasis						
H0	Reference			Reference		
H1	3.475	(2.733–4.417)	**<0.0001**	1.830	(1.279–2.618)	**0.001**
Tumor location						
A single part of the stomach	Reference			Reference		
Two parts of the stomach	2.049	(1.807–2.325)	**<0.0001**	1.156	(0.974–1.372)	0.098
The whole stomach	4.145	(3.421–5.022)	**<0.0001**	1.559	(1.187–2.047)	**0.001**
Borrmann type						
Type I	Reference			Reference		
Type II	0.574	(0.435–0.774)	**<0.0001**	0.877	(0.548–1.403)	0.584
Type III	1.286	(0.978–1.692)	0.072	1.186	(0.769–1.829)	0.439
Type IV	3.285	(2.449–4.407)	**<0.0001**	1.809	(1.137–2.877)	**0.012**
Histological type						
Well differentiated	Reference			Reference		
Moderately differentiated	1.586	(1.065–2.362)		1.076	(0.533–2.171)	0.839
Poorly differentiated	2.417	(1.648–3.545)		1.217	(0.610–2.427)	0.578
Pathological type						
Adenocarcinoma	1.024	(0.617–1.701)	0.927	0.787	(0.617–1.003)	0.053
Others	Reference			Reference		
T stage						
T1	Reference			Reference		
T2	1.788	(1.201–2.662)	**0.004**	1.328	(0.725–2.433)	0.359
T3	5.336	(3.930–7.246)	**<0.0001**	2.554	(1.468–4.443)	**0.001**
T4	10.898	(8.018–14.811)	**<0.0001**	3.074	(1.732–5.456)	**<0.0001**
pTNM						
I	Reference			Reference		
II	2.976	(2.107–4.203)	**<0.0001**	1.071	(0.637–1.801)	0.797
III	6.548	(4.736–9.052)	**<0.0001**	1.726	(1.001–2.976)	**0.050**
IV	23.195	(16.720–32.176)	**<0.0001**	3.899	(2.236–6.796)	**<0.0001**
N stage						
N0	Reference			Reference		
N1	3.195	(2.764–3.693)	**<0.0001**	1.595	(1.278–1.991)	**<0.0001**

Statistically significant results are shown in bold font. Reference: hazard ratio = 1.0.

The bold values means significant meanings.

### 3.4 Borrmann type IV and old age worsen the 5-year overall survival of female and T2 patients, respectively

The 5-year OS is a crucial metric for evaluating the effectiveness of GC surgery. Peritoneal metastasis, hepatic metastasis, tumor invading the whole stomach, Borrmann type III/IV, invasive depth, poor pathological stage, and lymph node metastasis were all identified as independent predictors of poor OS and 5-year OS (**Table 4**). Interestingly, the gender of females started to pose risks in the PAS group (OR = 0.983, 95% CI = 0.723–1.336 *vs*. OR = 1.277, 95% CI = 1.028–1.586, **Table 2**). The same goes with T2 (OR = 1.545, 95% CI = 0.499–4.775 *vs*. OR = 2.227, 95% CI = 1.124–4.271, **Table 2**). The proportion of Borrmann type IV significantly rose in the female subgroup, making the gender of women a risk factor (6.0% *vs*. 17.4%, *P* < 0.0001, **Table 5**). A higher percentage of elderly patients and hepatic metastases in the T2 stage subgroup might be responsible for the unanticipated change in T2 (39.0% *vs*. 48.7%, *P* < 0.0001, 0.0% *vs*. 0.6%, *P* = 0.0022, respectively, **Table 6**).

**Table 4:** Comparison of 5-year overall survival characteristics between the PRE group and the PAS group.

5-year overall survival	1994–2006 (PRE group)			2007–2019 (PAS group)		
Characteristics	Odd ratio	95% confidence interval	*P*-value	Odd Ratio	95% confidence interval	*P*-value
Age						
<40	1.528	(0.918–2.547)	0.108	1.305	(0.892–1.937)	0.18
40–49	1.671	(1.058–2.630)	**0.028**	1.3	(0.942–1.791)	0.116
50–59	Reference			Reference		
≥60	1.403	(0.977–2.013)	0.06	1.418	(1.111–1.818)	**0.006**
Gender						
Male, *n* (%)	Reference			Reference		
Female, *n* (%)	0.983	(0.723–1.336)	0.938	1.277	(1.028–1.586)	**0.031**
Hospitalization						
≤8, *n* (%)	Reference			Reference		
9–15, *n* (%)	0.795	(0.374–1.725)	0.571	1.407	(1.104–1.790)	**0.006**
>15, n (%)	1.157	(0.524–2.624)	0.725	2.31	(1.673–3.172)	**<0.0001**
Peritoneal metastasis						
P0	Reference			Reference		
P1	8.752	(4.353–17.597)	**<0.0001**	9.731	(6.128–15.453)	**<0.0001**
Hepatic metastasis						
H0	Reference			Reference		
H1	27.302	(3.735–199.587)	**<0.0001**	5.176	(2.110–12.693)	**<0.0001**
Tumor location						
A single part of the stomach	Reference			Reference		
Two parts of the stomach	2.258	(1.541–3.327)	**<0.0001**	2.784	(2.132–3.644)	**<0.0001**
The whole stomach	18.9	(5.128–79.91)	**<0.0001**	8.255	(4.499–14.900)	**<0.0001**
Borrmann type						
Type I	1.343	(0.718–2.629)	0.375	2.748	(1.458–5.228)	**0.0016**
Type II	Reference			Reference		
Type III	2.773	(1.922–3.990)	**<0.0001**	3.577	(2.666–4.800)	**<0.0001**
Type IV	17.16	(7.452–40.410)	**<0.0001**	17.09	(10.79–26.53)	**<0.0001**
Histological type						
Well differentiated	Reference			Reference		
Moderately differentiated	1.846	(0.892–3.957)	0.096	13.12	(2.164–136.5)	**0.0005**
Poorly differentiated	8.247	(4.165–16.910)	**<0.0001**	30.85	(5.327–318.500)	**<0.0001**
Pathological type						
Adenocarcinoma	1.228	(0.7163–2.107)	0.4725	1.171	(0.7331–1.911)	0.5208
Others	Reference			Reference		
T stage						
T1	Reference			Reference		
T2	1.545	(0.499–4.775)	0.425	2.227	(1.124–4.271)	**0.017**
T3	8.699	(3.948–18.31)	**<0.0001**	11.89	(7.139–20.480)	**<0.0001**
T4	19.13	(8.919–40.05)	**<0.0001**	62.82	(34.18–116.8)	**<0.0001**
pTNM						
I	Reference			Reference		
II	5.847	(2.743–12.540)	**<0.0001**	3.664	(1.789–7.444)	**0.0002**
III	22.26	(9.332–49.940)	**<0.0001**	18.19	(9.585–35.80)	**<0.0001**
IV	94.02	(33.700–237.100)	**<0.0001**	166.9	(80.640–327.700)	**<0.0001**
N stage						
N0	Reference			Reference		
N1	7.022	(4.603–10.712)	**<0.0001**	5.918	(4.612–7.595)	**<0.0001**

Statistically significant results are shown in bold font. Reference: hazard ratio = 1.0. The hazard risk (HR) with a corresponding 95% confidence interval (CI) was calculated using a random-effects model.

The bold values means significant meanings.

**Table 5:** Trends of hazards of 5-year overall survival characteristics in the female group.

5-year OS	1994–2006		2007–2019		
Characteristics	Male	Female	Male	Female	*P*-value
Survival or death rate					
Death	62.2%	61.7%	43.3%	49.4%	n.s.
Age, years					
≥60	51.6%	33.7%	51.1%	38.4%	n.s.
Borrmann					
Type IV	9.7%	6.0%	11.1%	17.4%	**<0.0001**
Hospitalization, days					
>15	32.0%	38.2%	16.9%	8.4%	n.s.
Peritoneal metastasis					
P1	12.9%	18.3%	10.9%	12.2%	n.s.
Hepatic metastasis					
H1	5.4%	5.7%	2.0%	2.4%	n.s.
Tumor location					
A single part of the stomach	72.4%	67.9%	75.7%	71.3%	n.s.
Histological type					
Poor differentiation	68.4%	80.6%	70.0%	81.9%	n.s.
Pathological type					
Adenocarcinoma	64.5%	35.5%	64.5%	35.5%	n.s.
T status					
T4	54.4%	56.2%	16.9%	16.7%	n.s.
pTNM					
IV	25.8%	31.5%	19.8%	21.3%	n.s.
N stage					
N1	65.5%	65.3%	61.9%	66.5%	n.s.

Chi-square tests were used in the calculation.

n.s., not significant.

The bold values means significant meanings.

**Table 6:** Trends of hazards of 5-year overall survival (OS) characteristics in the T2 group.

5-year OS	1994–2006		2007–2019		
Characteristics	T1	T2	T1	T2	*P*-value
Survival or death rate					
Death	13.6%	19.5%	7.5%	15.4%	0.0453
Age, years					
≥60	54.2%	39.0%	38.2%	48.7%	**<0.0001**
Gender					
Female	40.7%	46.3%	37.3%	28.8%	0.0002
Hospitalization, days					
>15	72.9%	63.4%	12.7%	17.3%	0.0005
Peritoneal metastasis					
P1	0.0%	4.9%	0.5%	1.3%	0.0009
Hepatic metastasis					
H1	1.7%	0.0%	1.4%	0.6%	**0.022**
Tumor location					
A single part of the stomach	84.5%	80.0%	89.6%	90.8%	n.s.
Histological type					
Poor differentiation	52.7%	59.0%	57.3%	59.2%	n.s.
Borrmann					
Type IV	6.9%	4.9%	3.3%	2.7%	n.s.
Pathological type					
Adenocarcinoma	9.4%	19.8%	13.5%	19.0%	n.s.
pTNM					
IV	2.5%	7.4%	1.0%	3.8%	n.s.
N stage					
N1	11.9%	38.5%	16.5%	42.9%	n.s.

Chi-square tests were used in the calculation.

n.s., not significant.

The bold values means significant meanings.

### 3.5 Correlation between clinicopathological features

**Figure 3:**
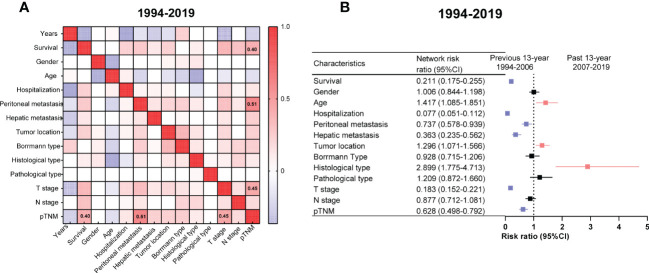
Correlation networks among the clinicopathologic characteristics of gastric cancer (GC) from 1994 to 2019 and relative risk (RR) of GC clinicopathologic characteristics between the PRE and PAS groups. **(A)** The correlation networks show different profiles of correlations among the clinicopathologic characteristics of GC from 1994 to 2019. Kendall’s tau-b was used for the correlation analysis. **(B)** The RR of clinicopathologic characteristics for GC. Symbols and error bars: black, *P >*0.05; blue, *P <*0.05 and RR <1; red, *P <*0.05 and RR >1.

The survival outcomes had positive correlations with pTNM (*r* = 0.40, **Figure 3A**). Undoubtedly, the pTNM presented a moderate correlation with peritoneal metastasis and the T stage in the graph (*r* = 0.51, *r* = 0.45 respectively, **Figure 3A**).

The patients in the PAS group tended to have older ages or poor differentiation. In contrast, the patients in the PRE group tended to have a worse prognosis, prolonged hospital stays, peritoneal or liver metastasis, deeper tumor infiltrating the gastric wall, and advanced cancer stages (**Figure 3B**).

### 3.6 Comparison of clinicopathologic features between two periods of 13 years

**Figure 4:**
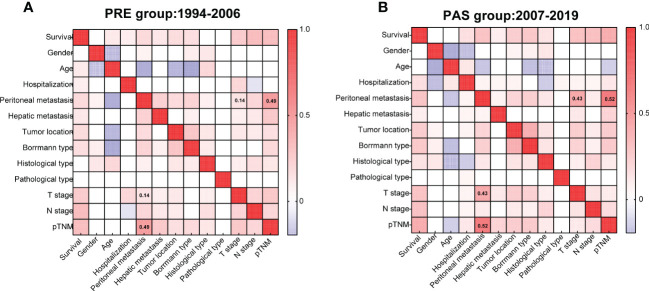
Heat maps showing the correlations of the gastric cancer patients’ clinicopathologic indices of the PRE group **(A)** and the PAS group **(B)**. Kendall’s tau-b was used for the correlation analysis.

**Figure 5:**
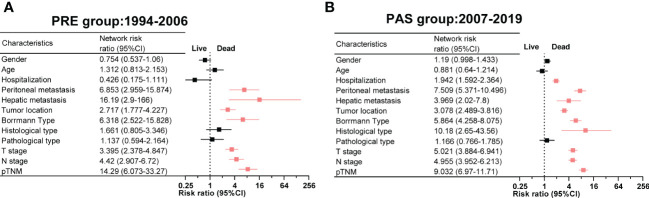
The relative risk of death and clinicopathologic features in the PRE group **(A)** and the PAS group **(B)**. Symbols and error bars: black, *P >*0.05; red, *P <*0.05 and RR >1.

Two heat maps show the correlations of GC patients’ indices. The correlation between peritoneal metastasis and T stage was becoming more significant in the PAS group (*r* = 0.14, **Figure 4A** *vs*. *r* = 0.43, **Figure 4B**). Hospitalization was shifting from being a protective factor in the PRE group to becoming a survival risk to those in the PAS group (RR = 0.426, 95% CI = 0.175–1.111, **Figure 5A** *vs*. RR = 1.942, 95% CI = 1.592–2.364, **Figure 5B**). The histological type was becoming more significant in threading patients’ lives (RR = 1.66, 95% CI = 1.592–2.364, **Figure 5A** *vs*. RR = 10.18, 95% CI = 2.65–43.56, **Figure 5B**). As time went on, hepatic metastasis reduced the risk factor for death (RR = 16.19, 95% CI = 2.9–166, **Figure 5A** *vs*. RR = 3.969, 95% CI = 2.02–7.8, **Figure 5B**).

## 4 Discussion

GC is the sixth most prevalent cancer worldwide and is always asymptomatic in behavior. GC symptoms imply the progression of the tumor. When cancer is detected at an early and asymptomatic stage, patients could have a greater chance of survival (15, 16). An endoscope is an effective tool for screening early lesions of the digestive tract. The 5-year survival rates of GC patients were reported approximately 90% in EGC after radical gastrectomy (17, 18). The proportion of EGC in the screened group was reported to be twice as high as that in the unscreened group (19–22). Patients had a better prognosis after EGC screening was popularized. Since Japan and Korea selected endoscopy screening as a population-based screening method in 2016, their GC mortality decreased, and the patients’ prognoses got better (10). According to the results of case–control research conducted in a community, endoscopic screening within 36 months of diagnosis reduced GC mortality by 30% compared with no screening (7). A large-scale, nested case–control study has demonstrated the efficacy of endoscopic screening in lowering GC mortality by comparing the data between individuals who received screening endoscopy and those who did not (OR: 0.53, 95% CI = 0.51–0.56) (13). The execution of endoscopy screening for GC is extremely beneficial for people over 50 years old at 2- or 5-year intervals (23, 24). Following the policy of EGC screening being approved in China in 2006, the First Affiliated Hospital, Sun Yat-sen University, conducted this screening in populations over 50 years of age in 2007 (25).

Our data have demonstrated that both the 5-year survival rate and the overall survival rate of patients in the PAS group significantly increased, and several characteristics had been altered, indicating the value of population-based EGC screening. The 5-year survival rates were 60.3% in Japan and 68.9% in South Korea from 2010 to 2014, while that was lower (52.0%) in South China when we incorporated GC diagnosis during 2010–2014 (14). The ECG screen may have contributed to the much higher survival rate (52.4%) in the PAS group compared with that in the PRE group (37.7%).

Previous research showed that Borrmann type IV was related to worse survival (26–28). Our study revealed that Borrmann type IV was more prevalent in females, which was discovered to be a risk factor for fatal 5-year OS outcomes in the PAS group. The absence of improvements in stage IV survival in the PAS group may be due to the higher prevalence of Borrmann type IV. Changes in the incidence of Borrmann type IV in the female or stage IV group have not been reported in any literature yet. The prognosis of patients with Borrmann type IV is believed to be poor due to more lymph node metastases, peritoneal metastases, serosal invasion, and lymphatic invasion (29, 30). The proportion of Borrmann type IV GC in female patients was reported as 10.3%–22.8%, which was consistent with our data (17.4%) in the PAS group but was higher than that in the PRE group (6%). For all patients with Borrmann type IV in this study, no significant difference was found between the PRE and PAS groups (12.0% *vs*. 12.8%). GC patients with Borrmann type I reported a better survival rate than those with other Borrmann types (31), while we found that Borrmann type II had the best survival contrarily. Our result was strongly supported by a previous study which showed that Borrmann type I GC had a significant rate of recurrence attributable to deeper serosal invasion and bigger tumor size (31).

Gender was considered to be related to GC characteristics (32). There were more male GC patients than female patients in our study (65% *vs*. 34%). However, the ratio of each gender remained constant, and our results showed no discernible variation in the prognosis of GC for either gender. A study pointed out that females tended to have better survival than males because of the lower grades and the smaller sizes of GC (32). Estrogens were proven to have a protective effect on GC in females since estrogens could regulate thyroid transcription factor proteins to protect the mucous epithelia or inhibit the expression of a c-erb-2 oncogene (33). Another meta-analysis showed that estrogens could decrease the risk of GC (34). Our study did not support the conclusion probably because of more Borrmann type IV cases in the female group. Racial differences should be considered when comparing with other races in the Surveillance, Epidemiology, and End Results Program (SEER) database (32). This SEER database consisted of 61,639 (63.8%) male and 34,862 (36.2%) female patients (32), which was the same as what Freddie Bray reported (63.7% *vs*. 36.3%) (35). The following results supported the gender proportion of GC in our data.

Even though age was not reported as an independent risk factor for the prognosis of GC (36), patients under the age of 40 had a worse prognosis (37–40). According to our study, these young GC patients had a worse prognosis than those between 50 and 59 years old. Many research works pointed out that inherited risk factors and *Helicobacter pylori* infection were associated with gastric carcinogenesis below 40 years of age and led to a poor prognosis (36, 41–43). In the PAS group, there were fewer young GC patients under the age of 40 (11.6% *vs*. 8.5%). The reduced ratio of young age in our data may be explained by the EGC screening for the population over 50. In addition, aged patients who were over 60 years had the poorest prognosis in the present study. The GC mortality of old patients (≥60 years) increased, especially in males (44). It was noted that patients over 60 had an extremely worst prognosis for GC, which was in line with what our investigation revealed. Age-related accumulation of DNA hypomethylation errors is strongly correlated with cancer (45). In this study, the increased proportion of elderly patients explained the risk factors that led to the T2 subgroup’s development as well as the N1 group’s unaltered outcome. There were no statistical differences between the proportion of ages 50 to 59 years in the PAS group (77.3%) and the PRE group (71.2%). In the current dataset, patients between the ages of 50 to 59 years had the best prognosis; however, if we classified these patients into a younger group and an older group, we would not be able to evaluate the survival characteristic of this group. Our multivariate analysis found that patients between the ages of 50 to 59 years had a better prognosis, which has not been reported in any literature yet. Generally, these patients aged 50 to 59 years had a stronger immunity than older patients aged over 60 years. Our further study indicated that patients between the ages of 50 to 59 years had a lower proportion of Borrmann type IV GC than those in the younger subgroups. Perhaps these were the reasons for the better outcomes of these patients aged 50–59 years.

Another crucial prognostic indicator is the T stage, which quantifies the degree of stomach wall invasion (46, 47). We observed a decrease in the T4 stage in the PAS group, which has a higher association with peritoneal metastases than other T stages. Along with EGC screening, a tumor might be diagnosed before it invades the stomach’s serosal surface or even neighboring tissues (T4). T2 is considered an unthreatening characteristic in GC clinically (38, 48, 49). However, the increased percentage of hepatic metastases in the PAS group was the risk that made the T2 subgroup hazardous. Hepatic metastasis, one of the most common ways of GC metastasizing anatomically, was supposed to be a poor factor for GC prognosis. In our data, hepatic metastasis is less in the PAS group (**Table 1**) compared with the PRE group, but not in the T2 subgroup. The substantially reducing hazard of hepatic metastasis demonstrated how this risk was likely diminished by the advancements in medical technology. Several clinical trials have achieved inspiring progress in adjuvant treatment, such as the SPIRITS trial, G-SOX trial, ToGA trial, and ATTRACTION-2 trial (50–53). Adjuvant treatment, including combined chemotherapy, targeted therapy, and immunotherapy, could improve the median overall survival of unresectable advanced GC patients by about 1.2–2.7 months compared with traditional chemotherapy. Moreover, radiofrequency ablation for liver metastasis of GC helped patients achieve satisfactory and better short-term outcomes (54–56). However, the prognosis of the patients with hepatic metastasis was not significantly improved, indicating that hepatic metastasis of GC needs more effective treatment.

The peritoneum is another typical organ of GC metastasis. Based on Stephen Paget’s hypothesis of “seed-and-soil”, peritoneal metastasis of GC happens through several procedures: penetration towards the serosal layer, seeding, and adhesion of tumor cells to the peritoneum, survival, and invasion through the basement membrane to subperitoneal tissue (57, 58). This provides some clues to explain the correlation between T4 and peritoneal metastasis. The 5-year OS of peritoneal metastasis was quite low (8.8% and 12.0% in the PRE and PAS groups, respectively), which meant more attention should be paid to treating peritoneal metastasis.

Encouragingly, the lower percentage of stage IV in the PAS group may be attributed to EGC screening. However, the prognosis for patients in stage IV was practically the same in the two periods because more patients with Borrmann type IV were found in the PAS group (**Table 2**). This suggested that clinicians and investigators should put more emphasis on the treatment of stage IV and Borrmann type IV GC.

A total of 2,804 patients in our data could be an epitome on behalf of the patients in South China. Compared with the patients of the northwestern region in China, the patients in South China were inclined to have less Borrmann type IV (12.5% *vs*. 20.1%), less tumor invasion of the whole stomach (5.2% *vs*. 7.4%), less lymphatic metastasis (63.0% *vs*. 84.1%), and less peritoneal metastasis (12.3% *vs*. 54.9%) (11). Although that study did not provide the survival information of the patients in northwest China, more carcinoma metastasis involved suggested some clues to a worse prognosis. Upon exploring the reasons, we found that the GC patients in that study were from a small region of Northwestern China, where a lower percentage of Han people were found (59.3% of GC patients were from ethnic minorities, predominantly Muslims) and probably had relatively unhealthy dietary habits. In contrast, 98.6% of the patients in our research were from the Han ethnic group. Genomic differences do exist between races but not between various ethnic nationalities (59, 60). Then we found patients in diverse regions had diverse dietary habits (61). The northwesterners enjoy drinking strong boiled brick-tea, which is processed by fungal growth. The author of that literature speculated that the fungi in the tea might have a relationship with the GC. Additionally, the northwesters prefer high-fat, fried, and salty foods, which are positively correlated with GC risk factors (62). According to a study of the dietary inflammatory index, these foods increased the risk of GC because they contained dietary salt, saturated fat, and trans-fatty acids (63, 64). Therefore, the distinct pathologic characteristics between south and northwest China may have been caused by regional food practices rather than minorities.

This study also has some limitations: firstly, this was an analysis based on a single hospital design, which was not able to avoid uncertain statistical biases; secondly, there was no test record of *Helicobacter pylori* infection, which has been positioned as a class of carcinogenic factors, and finally, some follow-up information was missing.

## 5 Conclusion

In summary, EGC screening may have contributed to the dramatic improvement in the prognosis of GC patients. However, no enhancement in the survival rate of the GC patients with lymphatic metastasis and stage IV may be attributed to the increased percentage of old patients (≥60 years) and Borrmann type IV, respectively. One of this study’s main objectives was to compare the OR of 5-year OS between the two 13-year periods. We observed that Borrmann type IV or old age was the risk of the female subgroup or the T2 subgroup, respectively. Other results indicated that the danger of hepatic metastases had diminished and that prolonged hospital stays and poor differentiation had turned into risks. Age, Borrmann type IV, and poor differentiation, therefore, continued to limit the longevity of GC patients, which calls for greater attention in terms of treatment.

## Data availability statement

The original contributions presented in the study are included in the article/**Supplementary Material**. Further inquiries can be directed to the corresponding authors.

## Author contributions

Writing original draft and formal analysis: HW and X-YZ; data interpreting: HW and X-YZ; data curation: HW, WL, and CD; resources: ZH; methodology: HC; project administration: YH, CZ, and MY; writing, reviewing and editing, and funding acquisition: XL; supervision, project administration, and funding acquisition: XJ. All authors contributed to the article and approved the submitted version.
