# Supplementary figures and images for: Gastric cancer clinical characteristics and their altered trends in South China: An epidemiological study with 2,800 cases spanning 26 years
Source: Front Oncol. 2023 Feb 7;13:976854. doi: 10.3389/fonc.2023.976854 (PMC9942704; doi:10.3389/fonc.2023.976854)

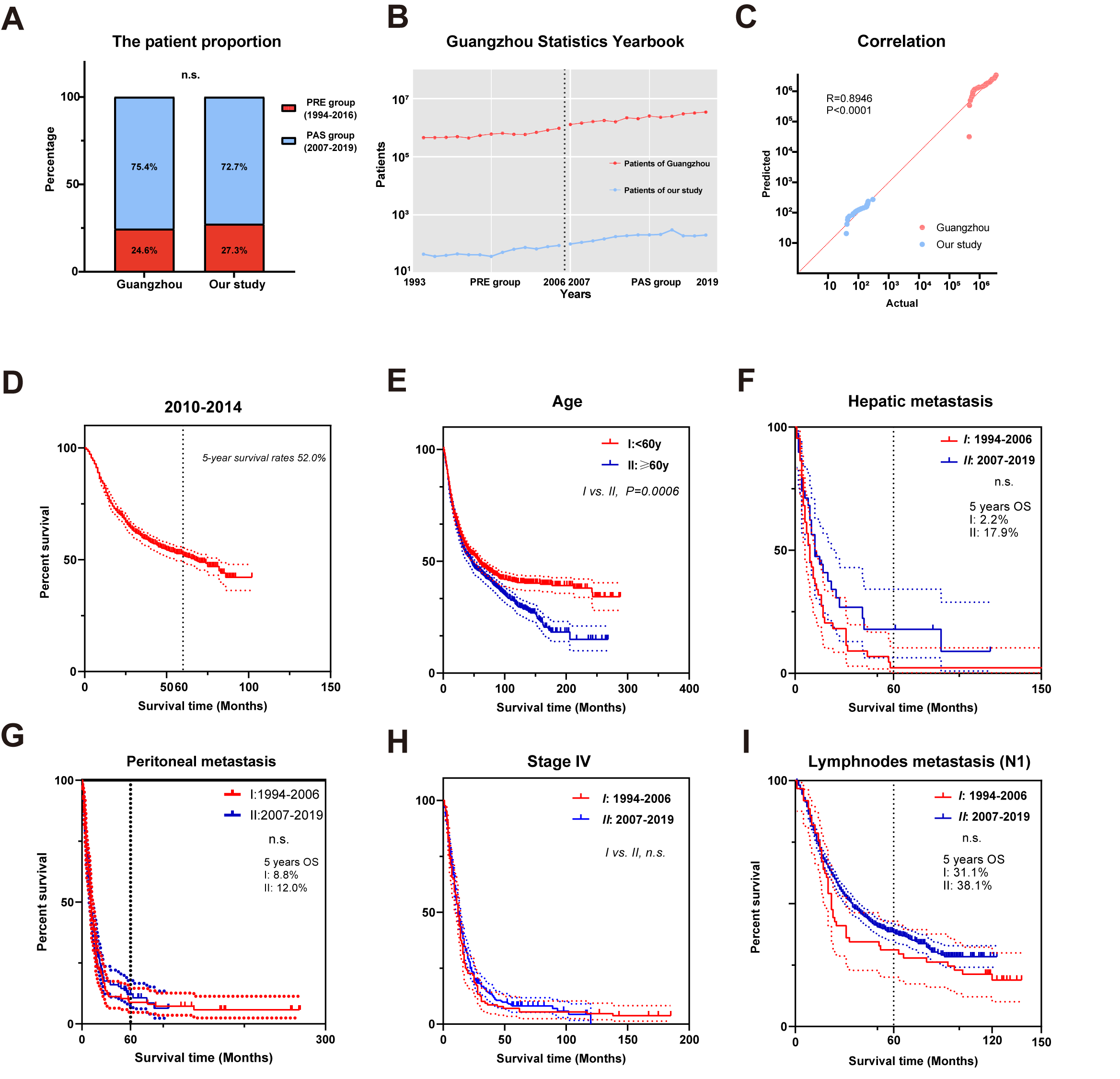

Supplement: Supplementary file 1 [file Image_1.tif]

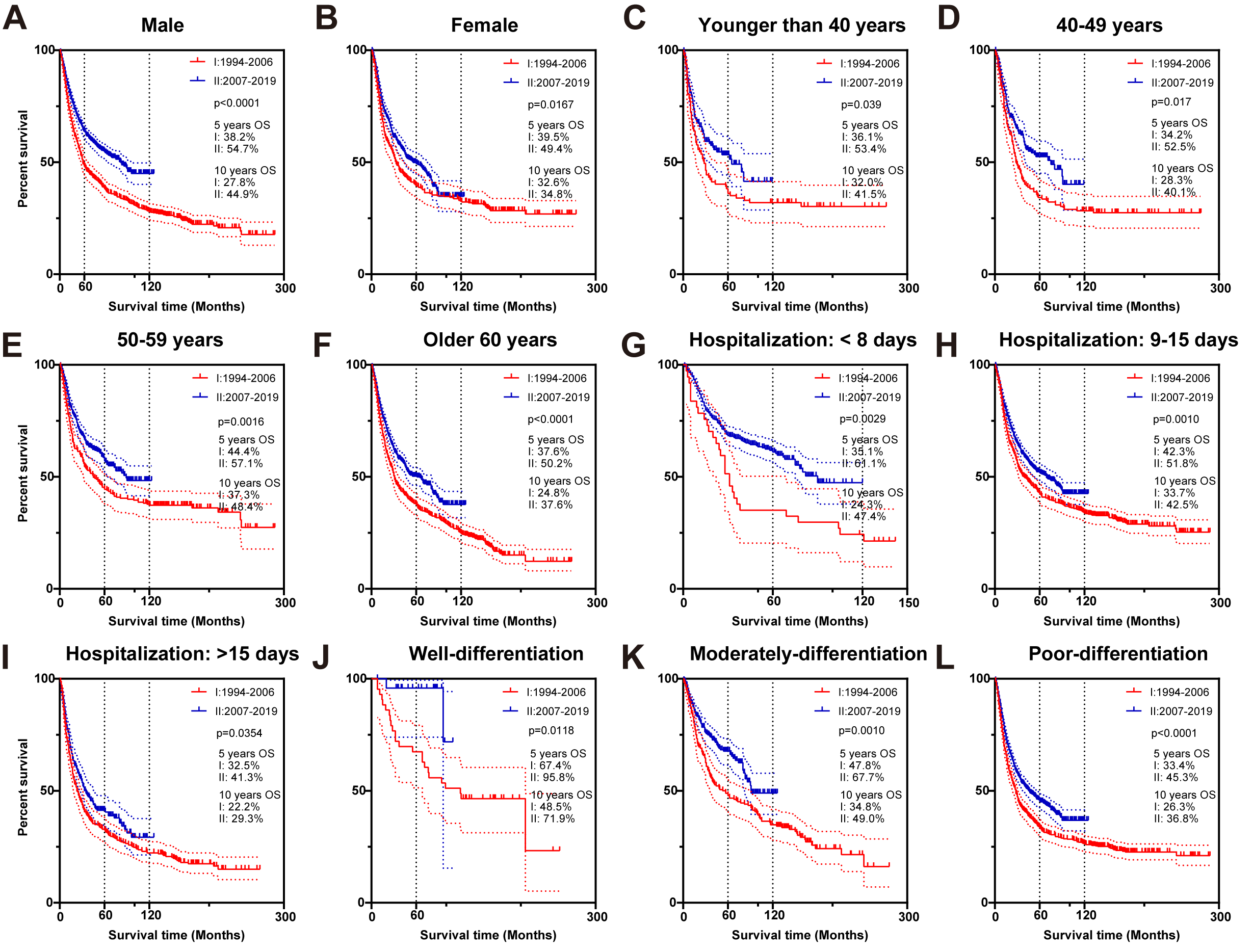

Supplement: Supplementary file 2 [file Image_2.tif]

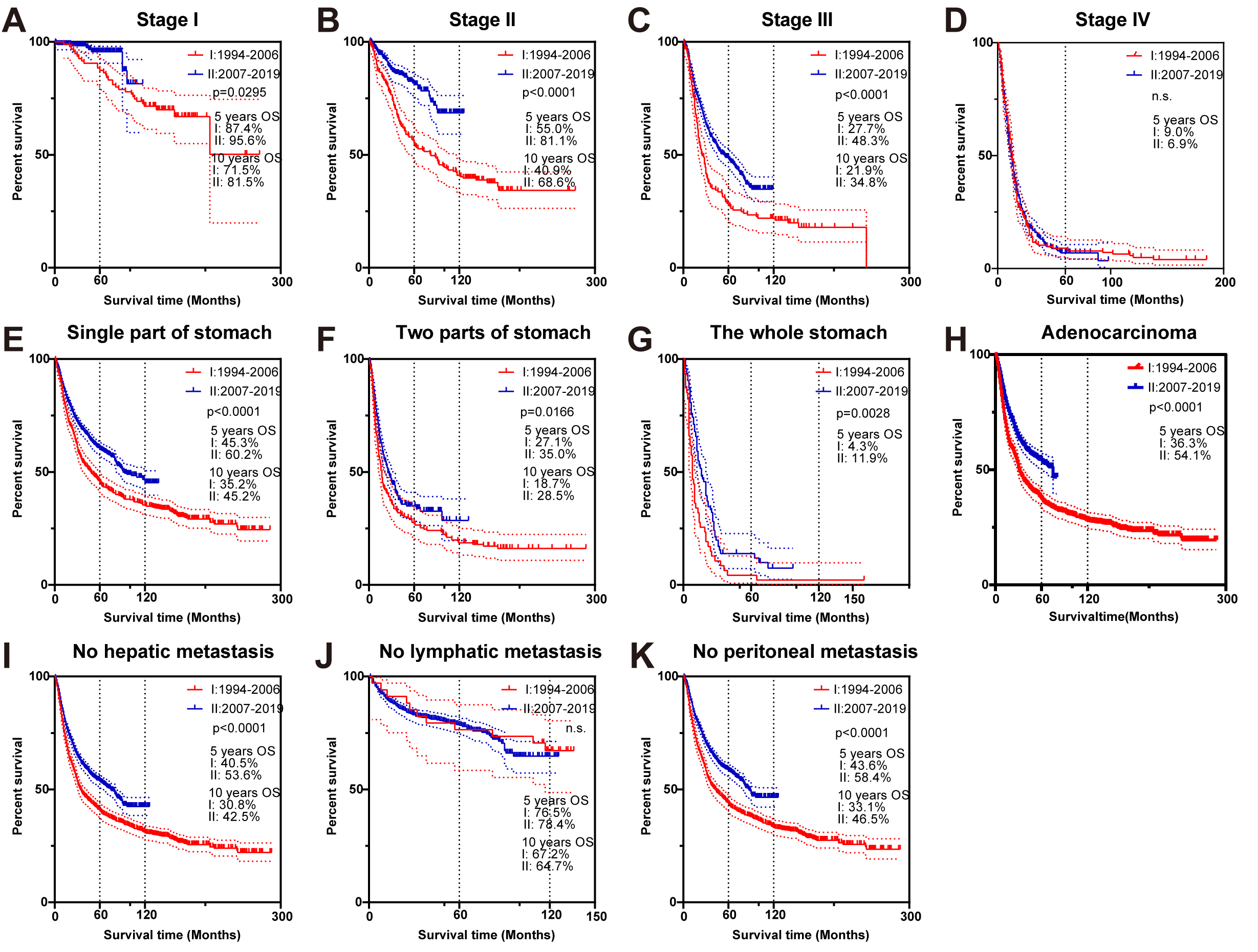

Supplement: Supplementary file 3 [file Image_3.tif]
