# Supplementary material for: Gastric cancer clinical characteristics and their altered trends in South China: An epidemiological study with 2,800 cases spanning 26 years
Source: Front Oncol. 2023 Feb 7;13:976854. doi: 10.3389/fonc.2023.976854 (PMC9942704; doi:10.3389/fonc.2023.976854)
Supplement: Supplementary file 4 [file Table_1.docx]

**Supplemental Table 1**

| **Age** | **Borrmann type** | | | | | | | |  |
| --- | --- | --- | --- | --- | --- | --- | --- | --- | --- |
|  | **Type I** | | **Type II** | | **Type III** | | **Type IV** | | **P-value** |
| <40 | 9 | (3.8%) | 54 | (22.5%) | 137 | (57.0%) | 40 | (16.7%) |  |
| 40-49 | 10 | (2.5%) | 107 | (27.5%) | 203 | (51.7%) | 72 | (18.3%) |  |
| 50-59 | 27 | (3.8%) | 174 | (24.7%) | 420 | (59.6%) | 84 | (11.9%) | 0.0022* |
| >60 | 61 | (5.0%) | 279 | (22.6%) | 764 | (62.1%) | 127 | (10.3%) |  |

*Patients aged 50-59 years were compared with younger-aged subgroups in Borrmann type IV.

Chi-squared tests are used to calculate.
